# Complex relationship among vessel diameter, shear stress and blood pressure controlling vessel pruning during angiogenesis

**DOI:** 10.1371/journal.pcbi.1013565

**Published:** 2026-01-12

**Authors:** Vivek Kumar, Prashant Kumar, Takao Hikita, Mingqian Ding, Yukinori Kametani, Masanori Nakayama, Yosuke Hasegawa

**Affiliations:** 1 Center for Research on Innovative Simulation Software, Institute of Industrial Science, The University of Tokyo, Tokyo, Japan; 2 Department of Mechanical Engineering, School of Advanced Engineering, University of Petroleum and Energy Studies (UPES), Dehradun, Uttarakhand, India; 3 Office of Innovative Medicine, Organization for Research Strategy and Development, Okayama University, Okayama, Japan; 4 Max Planck Institute for Heart and Lung Research, Bad Nauheim, Germany; 5 Department of Mechanical Engineering Informatics, School of Science and Technology, Meiji University, Kanagawa, Japan; Lehigh University, UNITED STATES OF AMERICA

## Abstract

Blood vessel pruning during angiogenesis is the optimization process of the branching pattern to improve the transport properties of a vascular network. Recent studies show that part of endothelial cells (ECs) subjected to lower shear stress migrate toward vessels with higher shear stress in opposition to the blood flow for vessel regression. While dynamic changes of blood flow and local mechano-stress could coordinately modulate EC migration for vessel regression within the closed circulatory system, the effect of complexity of haemodynamic forces and vessel properties on vessel pruning remains elusive. Here, we reconstructed a 3-dimentsional (3D) vessel structure from 2D confocal images of the growing vessels in the mouse retina, and numerically obtained the local information of blood flow, shear stress and blood pressure in the vasculature. Moreover, we developed a predictive model for vessel pruning based on machine learning. We found that the combination of shear stress and blood pressure with vessel radius was tightly correlated to vessel pruning sites. Our results highlighted that orchestrated contribution of local haemodynamic parameters was important for the vessel pruning.

## Author summary

Blood vessel networks formed by angiogenic vessel growth subsequently undergo extensive vascular remodeling process by regression of selected vascular branches. Optimization of the branching pattern in the vasculature is critical to ensure sufficient blood supply throughout the entire tissue. Recent studies have highlighted a strong relationship between the vessel remodeling and the shear stress acting on the vessel wall. However, its detailed mechanisms remain elusive due to the difficulties of estimating local haemodynamic parameters and relating them to vessel remodeling. Here, we have numerically simulated local haemodynamic parameters within the vascular network of the postnatal day 6 (P6) mouse retinal vasculature. Then, the relationship among the local shear stress, blood pressure, and vessel radius with the vessel pruning was examined. Moreover, we developed a predictive model for the vessel pruning based on the local haemodynamic parameters by a machine learning technique. Importantly, our results indicate that the combination of shear stress and blood pressure with vessel radius is tightly correlated to vessel pruning sites. Given the ongoing clinical approach to suppress tumor growth via blood vessel normalization, our results provide important knowledge for developing future medicine such as nanomedicine based on drug delivery systems.

## Introduction

A closed circulatory system in our body is comprised of the heart and the vasculature and is essential for development and tissue homeostasis to efficiently distribute nutrients, gases, liquids, signaling molecules and circulating cells. The blood is pumped out from the heart to the entire body through a complex hierarchical vascular network composed of arteries, capillaries and veins, causing the complex distribution of blood pressure and shear stress. Endothelial cells (ECs) form the inner lining of the vasculature and control the vascular branching pattern and other steps of vascular wall assembly [1,2]. As excessive branching leads to uneven blood flow distribution in a tissue, optimization of the branching pattern in the vascular network after angiogenic vascular sprouting is critical to ensure sufficient blood supply throughout the entire tissue with a limited power consumption of the heart [3–7]. Extensive rearrangement of vessel connectivity and endothelial specialization occur to ensure appropriate remodeling and quiescence of ECs. Vessel pruning leads to the selective removal of superfluous vessels by EC migration and rearrangement, leading to optimal blood flow distribution with reasonable overall pumping power to drive the blood flow.

Recent studies have highlighted a strong relationship between the vessel remodeling and the shear stress acting on the vessel wall. It has been reported that ECs tend to migrate from vessels with low shear stress to those with higher shear stress in opposition to the blood flow [3,4,6–9]. In the zebrafish midbrain and the cranial division of the internal carotid artery on eye during development, vessel pruning driven by haemodynamic parameters is observed in the developing vasculatures, leading to gradual reduction of the vascular complexity [3]. Importantly, some of the pruning sites correspond to low shear stress regions which are predicted by numerical simulation [4,10–12]. In the mouse retina, endothelial Partitioning defective 3 (PAR-3) plays a critical role in endothelial sensitization to the wall shear stress. The loss of PAR-3 in ECs causes defected axial polarization induced by the shear stress [13], resulting in enhancement of the vessel pruning. In contrast, ECs subjected to low shear stress do not always migrate toward high shear regions [14]. In tumors, blood vessels are unevenly distributed with a chaotic pattern and often exhibit irregular branching [15,16]. Not all open vessels are perfused continuously, and the blood flow distribution may change within a few minutes and the flow direction could be reversed even in the same vessel [15]. Dynamic changes of blood flow should affect not only local shear stress but also other factors including blood pressure or geometry of vessels. However, the combined effects of local haemodynamic factors on vessel regression to vascular network optimization remain elusive.

Here, we have reconstructed three-dimensional (3D) vessel structures from 2D confocal images of the postnatal day 6 (P6) mouse retinal vasculature and numerically simulated blood flow to obtain local haemodynamic parameters such as wall shear stress and blood pressure within the vascular network. We further investigated the relationship among the local haemodynamic parameters including shear stress, blood pressure, and vessel radius with the vessel pruning. Moreover, we developed a predictive model for the vessel pruning based on the local haemodynamic parameters by leveraging machine learning techniques, and the established model was validated with test samples.

## Results

### Impact of vessel pruning on flow field behavior in the vascular network

**Fig 1:**
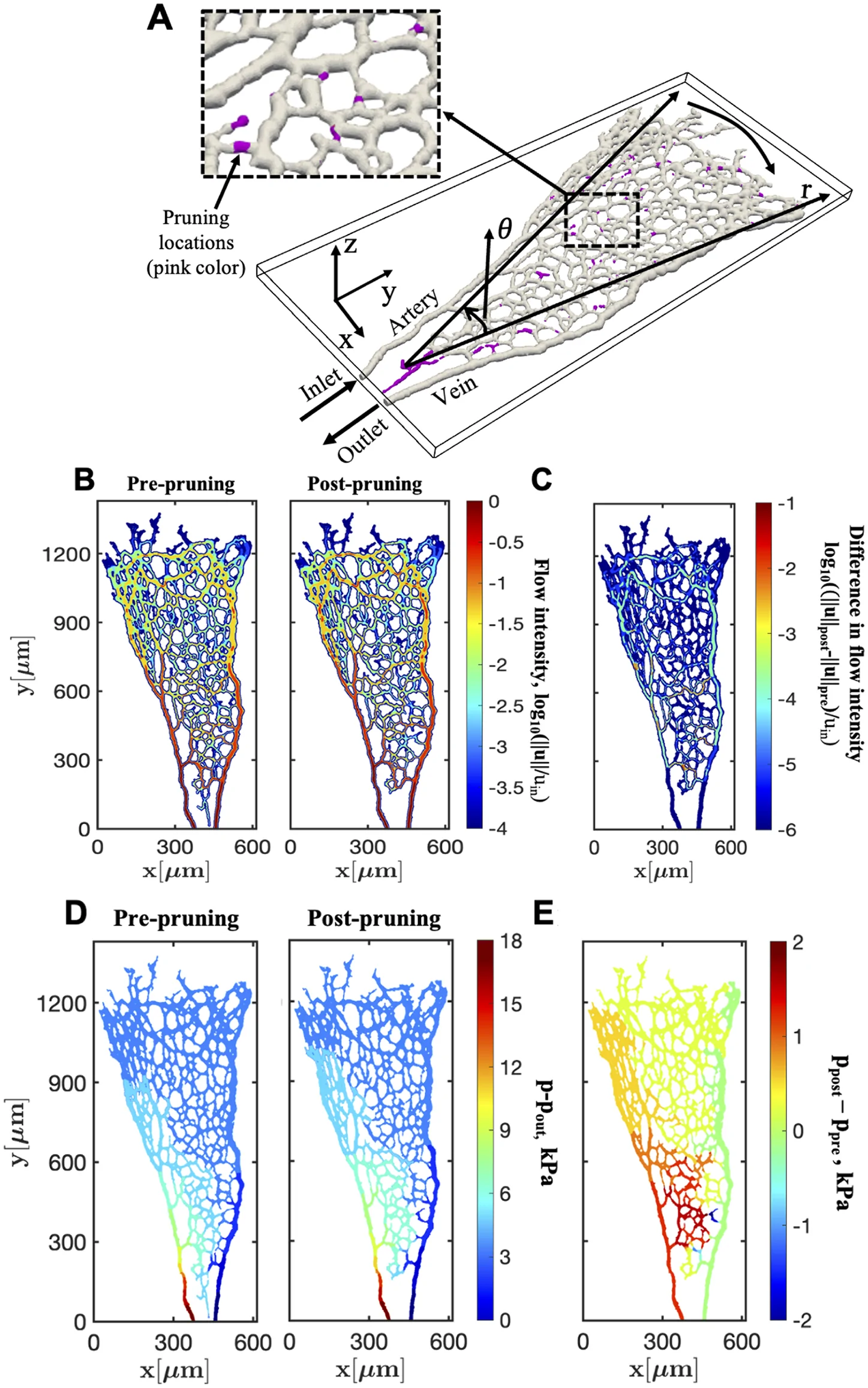
The effect of vessel pruning on haemodynamic parameters. **(A)** The 3D structure of the vascular network with pruned vessels. Gray: the structure after pruning and Pink: the pruned blood vessels. **(B)** Blood flow intensity of the pre-pruning and the post-pruning structures. Color scale represented the logarithmic normalized blood flow intensity (log_10_(‖***u***‖/*u*_*in*_)). **(C)** The difference in the flow intensities between the post- and pre-pruning structures in a logarithmic scale (log_10_((‖***u***‖_post_ - ‖***u***‖_pre_)/*u*_*in*_)). **(D)** Pressure distribution of the pre-pruning and the post-pruning structures. Color scale referred to the relative pressure to the outlet pressure, i.e., p-p_out_. **(E)** The difference between the pressures of the post- and the pre-pruning structures, i.e., p_post_ - p_pre_.

Angiogenic vascular growth is important to improve tissue hypoxia. To investigate whether vessel pruning contributes to the increase of blood flow at the angiogenic front, 3D vascular structures were reconstructed from 2D confocal images using an antibody against ICAM-II, the marker for the endothelial luminal surface as previously established [17]. Regressing ECs integrate into neighboring vessels and leave empty sleeves with the EC basement membrane, including collagen type IV as a remnant of the existed vascular connection [18–22]. The post-pruning structure corresponds to the vascular structure when a mouse eye was collected and it was reconstructed from the region where ICAM-II was positive. Meanwhile the pre-pruning structure was inferred by adding empty sleeves characterized by Collagen IV positive and ICAM-II negative region to the post-pruning structure. The added pruned vessels (empty sleeves) are shown in pink color (Fig 1A). The blood is supplied from an artery at the center of the retina and is distributed to the azimuthal direction through branching capillary networks to the angiogenic front where ECs are subjected to VEGF signaling, then flows into a draining vein toward the outlet. The spatial distribution of the blood flow can be considered as a key quantity to evaluate the transport properties of the vasculature. Blood flow was numerically simulated with the pre-pruning and post-pruning blood vessel networks and the obtained blood flow intensity in each network are shown (Fig 1B). Blood flow intensity is defined as ||u|| = $\sqrt{u^{\text{2}}+v^{\text{2}}+w^{\text{2}}}$, where *u*, *v* and *w* are the three velocity components of the blood flow. To examine the effects of the vessel pruning on the transport properties of the vascular network, the blood flow intensity of the pre-pruning structure was subtracted from that of the post-pruning structure. Blood flow around the angiogenic front was enhanced after vessel pruning (Fig 1C). The comparison of the pressure distributions between the pre-pruning and post-pruning vascular networks were shown in Fig 1D. In the present study, a zero pressure gradient has been applied at the inlet, so that a prescribed inlet blood velocity is realized. The outlet pressure was taken as a reference pressure and the Dirichlet boundary condition (p = 0) was imposed at the outlet in both structures, so that the plotted pressure represented the relative pressure from the outlet pressure. Furthermore, the difference between the local pressures in the post- and pre-pruning structures, i.e., p_post_ – p_pre_, was plotted in Fig 1E. The pressure was observed to be increased especially near the inlet after the vessel pruning. The ocular perfusion pressure (OPP) is the typical pressure difference that drives the flow in retina and it was reported approximately 57 mmHg [11,23]. This value for OPP was for 16 weeks old mice [23]. However, the present study was performed on mouse retinal vasculature of postnatal days 6 (P6). In our results, the pressure drop across the different networks were shown to be 135mmHg in Fig 1D and 65mmHg in S8B Fig. Meanwhile, the pressure drop in capillaries region was in the range of 20–30mmHg. Therefore, the pressure drop across the network may vary depending on their ages and each sample. Considering such variabilities due to the daily age of a mouse and the vessel region, the current results can be considered consistent with those in existing literatures.

### Effects of wall shear stress on the vessel pruning

**Fig 2:**
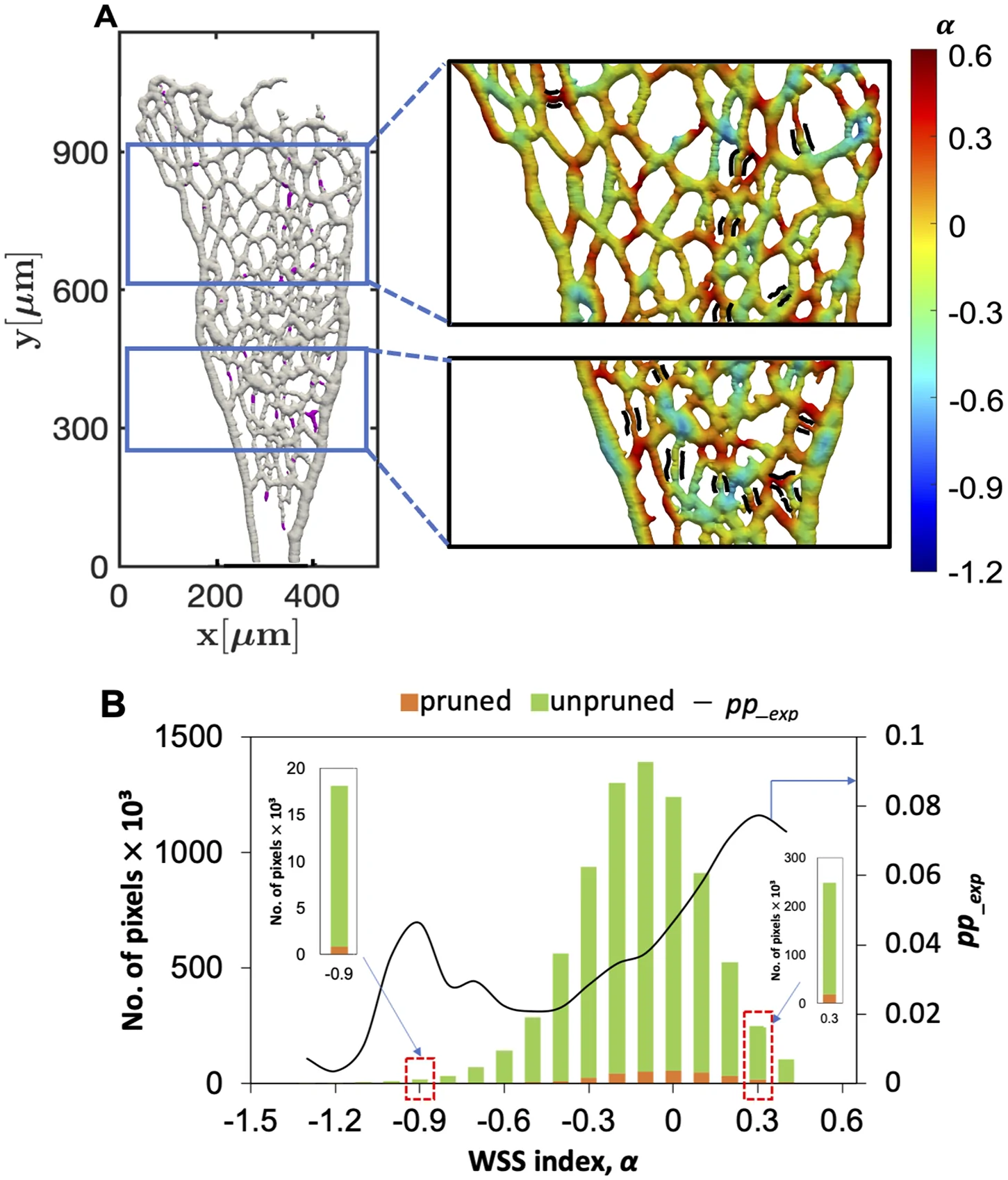
Effect of WSS on vessel pruning. **(A)** Actual pruning sites (pink) in the vascular network (gray) were shown in the left panel. Right panels showed *α* distribution in the vascular network. The color on the vessel surface represented the value of *α*, while the actual pruned vessels are bordered by black thick lines. **(B)** distribution of number of pixels (pruned and unpruned) and experimental pruning probability (*pp*__*exp*_) with *α*. Bars present number of pruned pixels (orange color) and unpruned pixels (green color), while solid black line presents the variation of experimental pruning probability (*pp*__*exp*_) with the *α*.

To gain further insights into the effects of vessel pruning on the transport property of the vasculature, we next analyzed wall shear stress (WSS) in several mouse retina samples (S2 Fig). The distribution of WSS was simulated as previously reported [17] and was compared with the locations of the pruned vessels (Fig 2A). The WSS obtained in the present simulation ranges from almost zero Pa to around 30 Pa, the range of which is reasonable comparing existing literatures [11]. Given the fact that EC subjected to low wall shear stress migrates toward to the region subjected to high shear stress [14], we aimed to visualize relative difference of local WSS. The local average of WSS (WSS_avg_) was calculated around each pixel within a local small window of 91.5 *µm* × 91.5*µm* × 36 *µm*. Then, a dimensionless WSS index (*α*) for the local shear stress is defined as ${\alpha =\text{log}}_{\text{1}\text{0}}\left(\frac{\text{WSS}}{{\text{WSS}}_{\text{avg}}}\right)$. To investigate the effect of window size on WSS_avg_, we have provided the distribution of experimental pruning probability (*pp*__*exp*_) over the range of *α* for different windows size (S3 Fig). When we increase the windows size from 91.5 *µm* × 91.5*µm* × 36 *µm* to 121.5*µm* × 121.5 *µm* × 36 *µm*, we did not observe significant change in *pp*__*exp*_. Specifically, the two distinct peaks at low and high WSS were commonly observed regardless of the window size. Therefore, we set the windows size as 91.5 *µm* × 91.5*µm* × 36*µm* in the present study. From the definition, *α* becomes positive when the local shear stress is larger than its average around that point, whereas it is negative for regions with lower shear stress. The contour of *α* in the pre-pruning vasculature was shown in Fig 2A. The range of *α* lies between -1.2 to 0.6. Since we are taking the logarithm of the ratio, it is not necessary that the distribution of *α* should be centered around zero. One discussion we could make from the asymmetric distribution of *α* is that the longer tail for a negative WSS indicates that there exist a substantial number of vessels with quite low flow rates and therefore low wall shear stress. This could also be observed the flow distribution in Fig 1B. The actual pruned vessel segments observed experimentally were bordered by thick black lines. Interestingly, not all of vessel branches with low WSS were regressed. Moreover, considerable amount of the vessels subjected to high WSS were also pruned (Fig 2A). We plotted the number of pruned (orange color) and unpruned (green color) pixels with the *α* (Fig 2B). Here, these pixels are those located at the surface of the vessel walls (the interface between the vessels wall and blood regions). We used all the seven samples (S2 Fig) to plot the Fig 2B. We next examined the experimental pruning probability (*pp_*_*exp*_ = pruned vessel wall pixels/total vessel wall pixels) obtained from the present experiment as a function of *α* (Fig 2B, solid black line). When splitting the entire range into low and high WSS regions as *α* < 0 and *α* > 0, it is found that the pruning probabilities are 0.031 and 0.065 for the ranges of *α* < 0 and *α* > 0, respectively. To get the pruning probability, we divided the number of pruned pixels for each category with the total number of pixels over all the seven samples (S1-S7). While the pruning probability had a peak at the low shear stress of *α* = −0.9, the highest peak was observed at *α* = 0.3 where the local shear stress was relatively high. The high pruning probability in the low WSS vessels have already been reported in previous studies [24,25], while the other peak in the high WSS vessels is first reported in the present study. We also note that large WSS can also be clearly observed in some pruned vessels in Fig 2A. Therefore, the large WSSs do not occur locally, but cover the entire pruned segments. In general, vessels with higher WSS tends to contribute to carrying more blood. Pruning such a large WSS vessel should suppress the functional shunt and draw blood to longer paths for better transport within the entire tissue. These results suggest the complex relationship between local WSS and vessel pruning.

### Development of a machine learning model to predict vessel pruning sites

**Fig 3:**
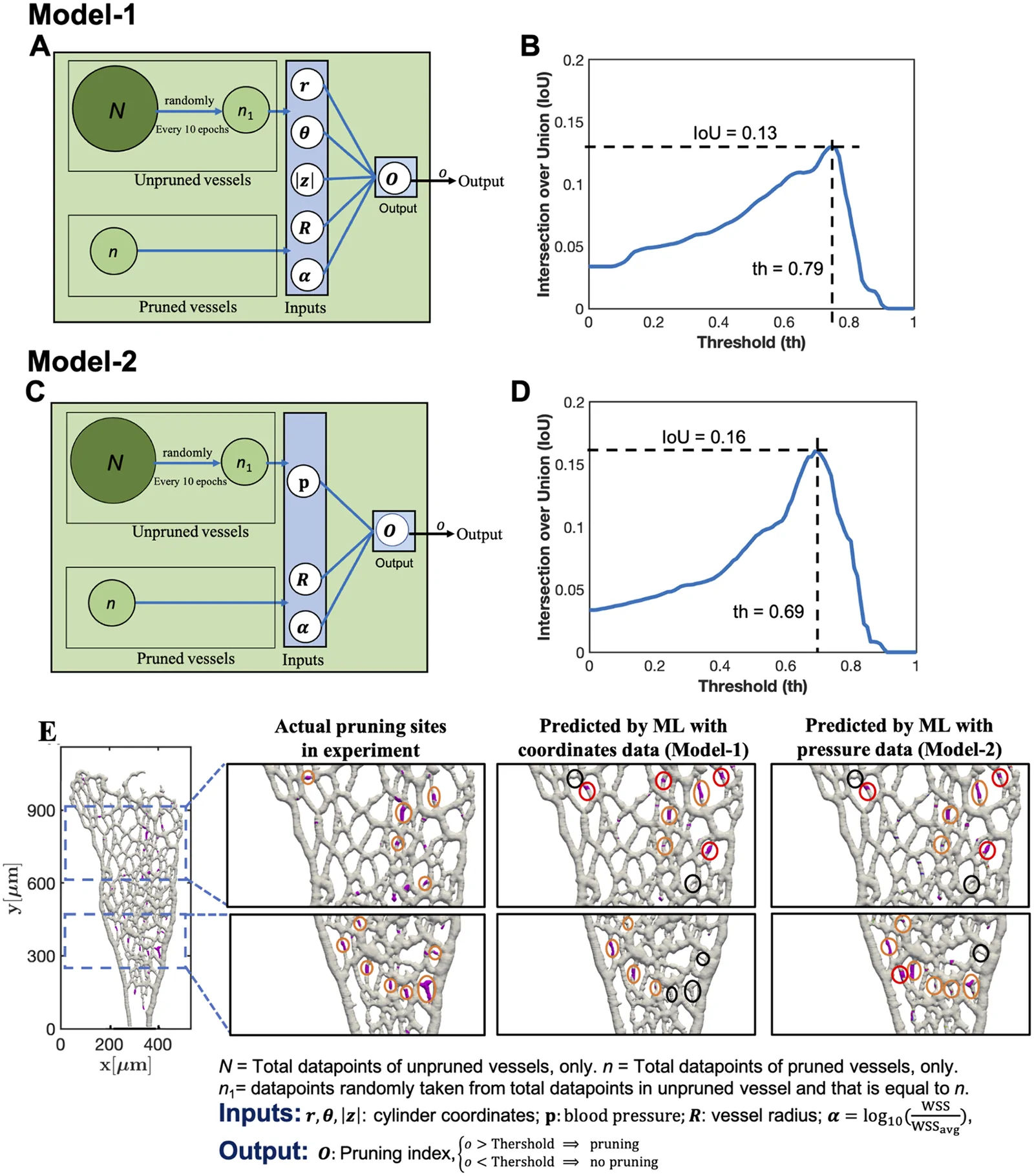
Vessel pruning prediction by machine learning model. **(A)** Schematic of the machine learning model (Model-1) in which the relative location from the inlet (r, θ, |z|), the vessel radius (*R*) and the *α* were input to the network, while the pruning index was output. *N* was the total number of pixels corresponding to unpruned vessels, while *n* was the total number of pixels corresponding to the pruned vessels. *n*_*1*_ was the number of pixels randomly sampled from *N* unpruned pixels every 10 epochs. **(B)** IoU as a function of the threshold value (th) of the pruning index output from Model-1. **(C)** Schematic of machine learning model (Model-2) in which blood pressure, vessel radius and WSS index were used as the input, and the pruning index was output. *N*, *n*_*1*_ and *n* were defined as same as in Model-1*.* **(D)** IoU as a function of the threshold (th) used in Model-2. (**E)** Comparison of vessel pruning predicated by machine learning models (Model-1 and Model-2) with the actual pruning sites. ML in the figure means ‘machine learning’. Orange, black and red circles illustrated the successfully predicted pruning sites, unpredicted pruning sites and extra predicted sites, respectively.

To further investigate the role of WSS on vessel pruning, we developed a machine learning model. Out of 7 samples (S1, S2, S3, S4, S5, S6 and S7) as shown in S2 Fig, 6 samples (S1-S6) were used for the training, whereas the remaining sample (S7) was used as a test data to validate the trained machine learning model. It was observed that pruning mostly occurred close to the inlet and outlet as well as around the angiogenic front near the vein. Considering the relative location of the pruning sites from the inlet and the outlet, we introduced a cylindrical coordinate system (*r*,*θ,z*) with its origin (reference point) at the first branching point from the arterial inlet as shown in Fig 1A. The radial and azimuthal directions were denoted by *r* and *θ* respectively, while |z| was the third dimension normal to the (*r* -*θ*) plane. Accordingly, as the inputs of the present machine learning model, we provided the relative location from the reference point, i.e., the coordinates: r,  θ, |z|, the local vessel radius (*R*) and *α* at the location of interest. Then, the model output the pixel-wise pruning index ranging between 0 and 1, where 0 and 1 indicate unprunned and prunned vessels, respectively. Hereafter, this model is referred to as Model-1. To effectively train the machine learning model, we randomly selected the same number of pixels from the unpruned regions as that of the pixels of the pruned ones (Fig 3A). During the training, we repeated randomly selecting the data points, i.e., the pixels from the unpruned vessels, every 10 epochs, while all the points of the pruned vessels were used for the training. As a result, our model predicted 9 pruned sites among 14 actual pruned vessels in the test sample S7. In addition, Model-1 incorrectly identified 6 pruning sites. The predicted pruning sites (pixels) were determined if the pruning index output from the model was greater than a critical value, which is determined by maximizing the Intersection over Union (IoU) between the prediction and the ground truth in the test sample. Specifically, the critical pruning index of 0.79 was used (Fig 3B). A vessel is defined as pruned if pixels identified as pruned completely cover the perimeter of the vessel. For more detailed procedures to determine the pruned vessels, see Materials and Methods.

### Local blood pressure information improves the success rate of the pruning site prediction

To investigate whether the vessel pruning can be predicted solely by local haemodynamic factors, we considered using the blood pressure instead of the coordinate information. We found that the blood pressure gradually decreased from the inlet to the outlet along the flow direction (Fig 1D) suggesting that the magnitude was implicitly related to the distance from the inlet. To incorporate this information of the local blood pressure, the machine learning model was modified. The coordinate information was removed from the input and the local pressure information was newly added in Model-2 (see, Fig 3C). From actual 14 pruning locations, Model-2 could successfully predict 11 locations, while Model-1 only predicted 9 locations. Model-2 also predicted less extra pruning locations, i.e., 4 locations, whereas 6 locations were incorrectly predicted by Model-1. Consequently, the success rate (i.e., the percentage ratio of predicted pruning sites and the total pruning sites) for predicting the pruning sites was increased from 64% to 78.5% and IoU was increased from 0.13 to 0.16 (Fig 3B and 3D). The critical pruning index of 0.69 was used for Model-2 (Fig 3D) to determine the maximum IoU between the prediction and the ground truth. We also calculated the critical pruning indexes for all the training samples and it varied between 0.67 to 0.79. Most unpredicted pruning sites in Model-1 were located at the vascular plexus around the center of retina, where *α* showed relatively high values (Fig 3E). In contrast, most of incorrectly predicted pruning sites existed around the angiogenic front in both models. To further verify the generality of the developed machine learning models (Model-1 and Model-2), we changed the combination of the training samples (S2-S7) and testing sample (S1) out of the total 7 samples (S1, S2, S3, S4, S5, S6 and S7) as shown in S2 Fig. The comparison of vessel pruning predicted by the machine learning models with real pruning sites observed in the experiment for the testing sample (S1) is shown in S5 Fig. Among actual 15 pruning locations, both Model-1 and Model-2 were able to predict 13 locations, resulting in the prediction success rate of 86.6%. However, Model-2 predicted less extra pruning locations (7 locations) as compared to Model-1 (8 locations). Intersection over Union (IoU) also increased from 0.19 to 0.21 (S5B Fig) by switching from Model-1 to Model-2. Hence, the superiority of Model-2 using the local blood pressure over Model-1 remained unchanged even when the training and testing data were changed.

### The combination of *α*, local blood pressure and vessel radius improved pruning prediction accuracy

**Fig 4:**
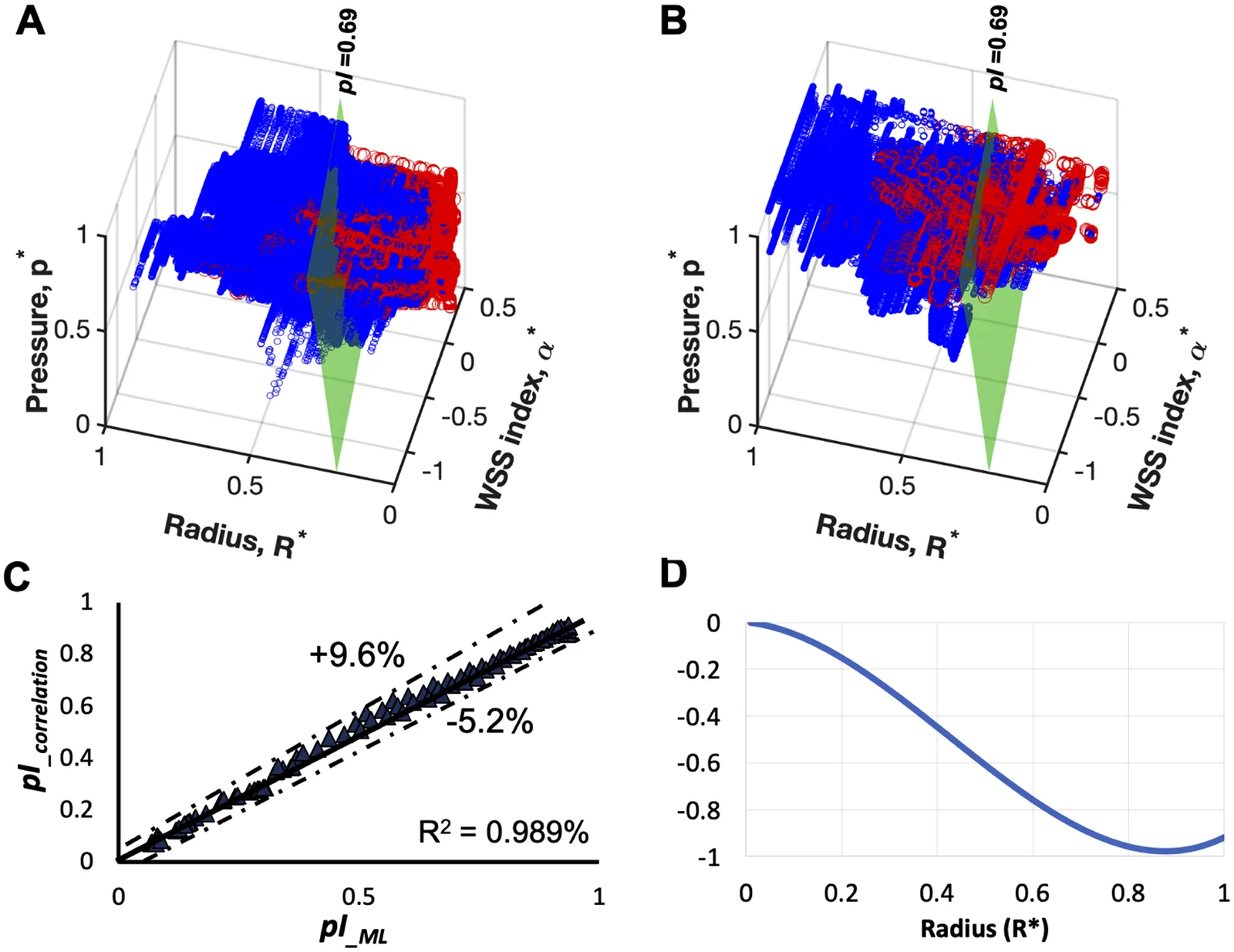
Evaluation of the model by the machine learning model with blood pressure. **(A and B)** Visualization of dependency of pruning probability on pressure, vessel radius and WSS index. The blue and red scattered circles showed unpruned and pruned pixels from experimental data **(A)** and prediction of the pretrained network **(B)**. The iso-surfaces illustrated the pruning index (*pI*) = 0.69 as green color) that predicted from pre-trained network. p* and R* indicated the scaled pressure and radius. α* referred to the scaled WSS index. **(C)** The variation of predicted pruning index by correlation with the pruning index by machine learning algorithm (*pp_*_*ML*_). **(D)** The contribution of the vessel radius to the pruning probability, i.e.,$\;\text{2}.\text{6}{\text{R}}^{*\text{3}}-\text{3}.\text{3}{\text{R}}^{*\text{2}}-\;\text{0}.\text{18}{\text{R}}^{*}\;$appearing in Eq. (1), as a function of the dimensionless vessel radius R^*^.

The input parameters in Model-2 were WSS index α^*^, local blood pressure p^*^ and local vessel radius R^*^. All these quantities were rescaled so that the range of the change for each variable was −1.3 ≤ α^*^ ≤ 0.3, 0 ≤ p^*^ ≤ 1.0, and 0 ≤ R^*^ ≤ 1.0, respectively, which was commonly used for efficient training of deep neural networks [26,27]. As mentioned in the previous section, we selected the same number of unpruned vessel points as that of pruned vessel for network training. Here, vessel points are the pixels consisting of the vessel walls, and for all the vessel wall pixels, the local input variables, i.e., WSS index α^*^, blood pressure p^*^ and vessel radius R^*^ are defined. In Fig 4A, the blue and red circles corresponded to unpruned and pruned pixels, respectively. We also plotted the iso-surface of the pruning index, *pI_*_*ML*_ = 0.69 estimated from the trained network of Model-2 with green color. It can be confirmed that the trained network captures the general trend of the training samples quite well, suggesting the training has been successfully conducted. To validate the generality of the trained network, the same plot has been provided for the test sample. The training network predicted the pruned vessel for the unseen data well (Fig 4B).

Furthermore, the iso-surface of the pruning index obtained by the trained network indicated that the pruning index increased with increasing the WSS and the blood pressure, and decreasing the vessel radius. The predicted pruning index from Model-2 as expressed in Eq. (5) is already simple, but the sigmoid function applied just before the output of the network makes the contribution of each term slightly unclear. Therefore, we correlated the output from the Model-2 with the following even simpler formula with the R-square value of 0.989:


$$\begin{array}{c} {pI\_}_{correlation}=\text{0}.\text{059}\alpha^{*}+{\text{0}.\text{089p}}^{*}+\text{2}.\text{6}{\text{R}}^{*\text{3}}-\text{3}.\text{3}{\text{R}}^{*\text{2}}-\;\text{0}.\text{18}{\text{R}}^{*}+\text{0}.\text{94}. \end{array}$$ (1)

We also fitted the pruning index of Model-2 with linear and different polynomial degree. S1 Table shows that the R-square value almost saturates at the third degree and further increasing the degree does not improve the fitting of the output of the model. Proposed correlation demonstrates that the radius has largest factor compared to pressure and WSS index. When considering a single vessel in a capillary network, if the pressure difference between its two ends is kept constant, the WSS of that vessel decreases proportionally with decreasing the vessel radius. This indicates that the vessel radius and WSS are strongly related to each other through haemodynamics. The significant increase of the pruning index with decreasing the vessel radius in Eqs. (1) and (5) corresponds to the finding in the previous studies that a vessel with lower WSS is more likely to be pruned. Indeed, as shown in Fig 2B, the current model also results in a peak of the pruning possibility at low WSS as in the previous studies. This is mainly attributed to the contribution of the vessel radius to the pruning index in the present study. In addition to the above-mentioned primary effect of the local radius, the present model suggests secondary contributions from WSS and blood pressure expressed by the first two terms in Eq. (1), though their contributions are relatively smaller than that of the vessel radius. This indicates that, among small vessels which tend to have relatively low WSS, vessels with relatively high WSS are more likely to be pruned. In order to clarify the importance of the secondary contributions from the haemodynamic parameters to the pruning prediction, we conducted further prediction of the pruning sites based on the local radius only, as shown in S6C Fig. In our original prediction model (Eq. 1) based on the local radius, WSS index and pressure, the number of correctly predicted, unpredicted and extra-predicted sites are 11, 3 and 4, respectively. They are respectively highlighted by orange, black and red in S6B Fig. When only the local radius is used as an input, the number of correctly predicted vessels is reduced from 11 to 9, while the numbers of unpredicted and extra-predicted sites are increases from 3 to 5 and 4 to 7, respectively. These wrongly predicted sites are highlighted by the same colors in S6C Fig. Furthermore, the circles with broken black and red lines in S6C Fig show unpredicted and extra predicted cites newly appear when only the local radius is used for the prediction. It is also confirmed that the newly emerged unpredicted cites have large relative WSS, while the extra predicted cites have low WSS (S6D Fig). According to Eq. (1), the pruning index is increased with increasing WSS, and this contributes to improve the prediction of the pruning. Therefore, even though the contributions of the haemodynamics in Eqs. (5) and (1) appear smaller than that of the vessel radius, we confirmed that the inputting the haemodynamic parameters in the machine learning model improves the pruning prediction.

To further assess the robustness and generalizability of our model, we conducted an additional analysis using 5 samples for the training (S1, S4, S5, S6, and S7) and 2 previously unused samples for the testing (S2 and S3). This new configuration allows us to validate the predictive performance of Model-2 with less training data and more unseen data. We compared the predictive performance of our original model based on the local radius, the pressure, and the wall shear stress against the model trained with only the local radius. The results indicate that the model incorporating all the three inputs outperforms the model based on only the local radius, indicating the importance of the hemodynamic parameters as the model inputs. Specifically, for sample S2, the model with all the three inputs correctly predicted 18 pruning sites, while 12 sites are unpredicted and 12 are extra-predicted. They are respectively highlighted by orange, black and red in S7B Fig. When only the local radius is used as an input, the number of correctly predicted vessels is reduced from 18 to 17, while the numbers of unpredicted and extra-predicted sites are increases from 12 to 13 and 12 to 15, respectively (S7C Fig). The newly added unpredicted and extra-predicted cites in the model with the single input are shown in S7C Fig with dotted black and red circles, respectively. For sample S3, the model with all three inputs correctly predicted 13 pruning sites, with 8 unpredicted and 24 extra-predicted sites (S7E Fig). Although using only the local radius as an input did not affect the number of correctly predicted and unpredicted sites, the number of extra-predicted sites increased from 24 to 27 (S7F Fig). Furthermore, the circles with broken red lines in S7F Fig show extra-predicted cites newly appear when only the local radius is used for the prediction. Therefore, even for the reduced number of training samples, our results confirm that incorporating haemodynamic parameters into the machine learning model improves pruning prediction accuracy.

To further address the effects of the number of training data on the model predictions, we also performed additional analyses by varying the number of training samples while the test sample is fixed. Specifically, we reduce a number of training samples from the original 6 samples down to 4 samples, while the test sample is fixed to S1. The results indicate that the model performance remains consistent across different numbers of training samples (S4B–S4D Fig), while the Intersection over Union (IoU) showed a marginal decline from 0.21 (with 6 training samples) to 0.20 (with 4 training samples). These results confirmed the generality of the present results even though the total number of samples are relatively small. This is partially because the current model predicts the pruning on a pixel-by-pixel, and a relatively large number of pixels are contained in each sample.

The pruning index by Model-2, *pI_*_*ML,*_ and the proposed correlation, *pI_*_*correlation*_ obtained from Eq. (1) were compared in Fig 4C. The proposed correlation provided satisfactory estimate of the output of Model-2 within the error between −5.2% to +9.6%. From Eq. (1), we can conclude that the higher WSS increased the pruning probability, since its proportional constant was 0.059, and positive. Similarly, a larger local pressure promoted more vessel pruning, and this trend was even stronger than that of WSS, as the proportional constant for the local pressure was larger. The contribution of the vessel diameter to the pruning probability, i.e., $\text{2}.\text{6}{\text{R}}^{*\text{3}}-\text{3}.\text{3}{\text{R}}^{*\text{2}}-\;\text{0}.\text{18}{\text{R}}^{*}$, was plotted as a function of the dimensionless radius (Fig 4D), indicating that the pruning probability decreases with increasing the vessel radius.

**Fig 5:**
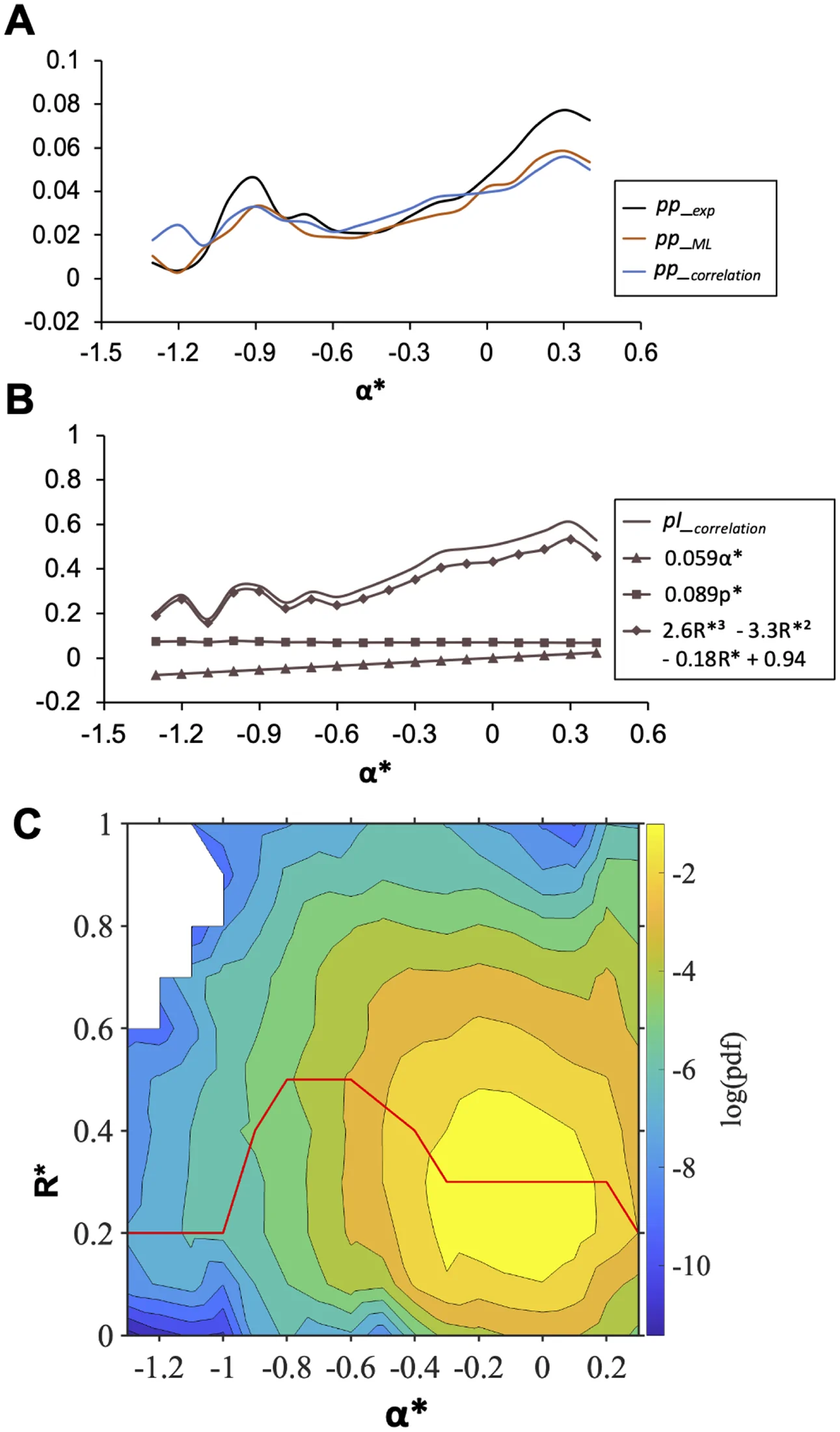
Contribution of haemodynamic factors on vessel pruning sites. **(A)** Comparison of pruning probability by correlation (*pp*__*correlation*_), pruning probability predicted by machine learning (*pp*__*ML*_) and experimental pruning probability (*pp*__*exp*_). **(B)** *pI*__*correlation*_ and their contributed factors were plotted against α^*^ with voilet lines with different solid symbols. **(C)** Joint probability density function (PDF) of the normalized WSS index α^*^ and vessel radius R^*^. The red line shows the maximum location of the joint PDF at each α^*^.

Next, we compared the experimental pruning probability with the pruning probability calculated from the model output and from the correlation. The pruning probability directly calculated from the model output (Eq. 5) with a proper threshold is referred as *pp*__*ML*_, while that calculated from the correlation (Eq. 1) is *pp*__*correlation*_. These pruning probabilities *pp*__*ML*_ and *pp*__*correlation*_ were plotted with the *pp*__*exp*_ as a function of WSS index (α*) (Fig 5A). *pp*__*ML*_ and *pp*__*correlation*_ have good agreement with *pp*__*exp*_. We note that the two peaks are underpredicted in both *pp*__*ML*_ and *pp*__*correlation*_. This is attributed to the fact that Fig 5A shows the pixel-wise pruning probability. On the other hand, the success rate is calculated for each vessel. Furthermore, we investigated the contribution of each haemodynamic factor on the pruning index (Fig 5B). According to Eq. (1), α* increased the pruning index linearly (Fig 5B, the violet solid line with triangles), whereas the experimental result showed a non-monotonic trend with respect to α* (Fig 5A, the black line). This discrepancy can mostly be explained by the contribution from R* which showed non-monotonic dependency on α*(Fig 5B, the violet line with diamonds). As a result, by summing up all the contributions, the non-monotonic dependence of the experimental data *pp*__*exp*_ can be predicted by the index (*pI*__*correlation*_) (Fig 5B, the violet line). To gain further insight into the contribution of R* and α* on *pp*__*correlation*_ their joint probability density function (PDF) was plotted and the most probable value of R* at each α* was shown by a red curve (Fig 5C). As smaller R* was seen at the vessels with both lower and higher α*, *pI*__*correlation*_ resulted in non-monotonic dependency on α* (Fig 5A).

## Discussion

Previous studies demonstrated the importance of WSS on vessel pruning [5]. During angiogenesis, vessel regression is induced by polarized endothelial cell migration. ECs subjected to low WSS are known to migrate toward the high WSS region for vessel pruning [4]. For polarized EC migration, dynamic changes of microtubules are observed with continuous blood flow [28,29]. EC shape is elongated along the axis of blood flow and migration. Microtubules are stabilized toward the direction of cell migration and the microtubule organization centers (MTOCs) and the Golgi apparatus are aligned in front of the nuclei toward the direction of migration. The loss of cell polarity factor, PAR-3 in ECs attenuates microtubules stabilization and polarization along the axis of blood flow [13]. As a result, vessel regression is compromised suggesting importance of WSS on determining vessel pruning sites in the vascular network [30]. However, we showed that only a limited fraction of ECs subjected to low WSS underwent vessel pruning. Moreover, the vessels even exposed to high WSS were often regressed (Fig 2B). To gain further insight into the role of mechano-stress on vessel pruning, we have developed two machine learning models to predict the pruning probability of the growing vasculature in the mouse retina. In addition to vessel diameter and local WSS, Model-2 configurated with the local blood pressure information, was compared to Model-1 which estimated the pruning probability based on the relative location of the point of interest from the inlet. As a result, Model-2 showed improved efficiency for the prediction, suggesting the importance of blood pressure during vessel pruning.

Consistently, recent studies have shown that blood flow promotes lumen formation by inducing the formation of inverse membrane blebs during angiogenesis [31]. Moreover, intraluminal pressure (IP) driven by blood flow is shown to be critical for wound angiogenesis in zebrafish and in vitro cell culture [32]. During wound angiogenesis, blood flow-driven IP loading inhibits elongation of injured blood vessels located at sites upstream from blood flow, while downstream injured vessels actively elongate. IP induces actin cytoskeletal reorganization in ECs under Rho family small GTPase, Cdc42 [32]. Since ECs are exposed to not only shear stress but also blood pressure, the orchestration of microtubules stabilization and actin cytoskeletal reorganization would be important for vessel pruning. Signaling crosstalk among Rho family small GTPases such as RhoA, Rac1 and Cdc42 is tightly controlled downstream of EC-to-EC junctions, integrins or primary cilia, modulating microtubule stabilization and actin cytoskeletal rearrangement [33–36]. Interestingly, cell polarity factors, such as PAR-3, aPKC or PAR-6 are known to mediate signaling crosstalk among RhoA, Rac1 and Cdc42 [34,37–39].

The present analysis focuses on time-averaged blood flow, and does not directly take into account the pulsating nature of cardiac cycles. When the pressure difference between the inlet and the outlet varies in time, it is important to consider how the blood flow and pressure react to it within the vasculature. For the mouse retina considered in the present study, the Reynolds number is quite low (~O(0.1)) and the Womersley number is also much smaller than unity. The former indicates that the non-linearity in the governing equation is minor, so that the flow dynamics can be considered linear. The Womersley number is interpreted as the ratio of the time required for the wall effects to propagate to the vessel center due to viscosity and the temporal pulsation cycle [40]. When the Womersley number is less than unity, the flow becomes quasi-steady, so that the pressure gradient almost balances with the viscous effects. In summary, when the pulsation is present, the blood flow, the wall shear stress and also the blood pressure inside the vasculature respond linearly. Hence, although the pulsation is not explicitly taken into account, the solutions under the present assumption of a steady flow possess the essential features of a pulsating flow. As far as the Reynolds number and Womersley numbers are sufficiently small, the present analyses can be used for other vasculatures. As for the mechanisms and the predictability of the pruning based on hemodynamics, it is unclear how the present model can be applicable, since the pruning mechanisms may differ among species and organs. Nonetheless, the present machine-learning-based framework for predicting pruning vessels can easily be applied to other vessels, so it is of interest to validate the generality of the current model, and the applicability of the present model to other organs or species in future work.

Next, we investigated the contribution of each haemodynamic factor on vessel pruning based on the machine learning model incorporated with blood pressure information. The pruning probability did not show monotonic correlation with WSS and have two peaks at both lower and higher WSS (Figs 2B and 5A). According to Eq. (1), the contribution of vessel diameters on pruning was larger than that of WSS, thereby elucidating the non-monotonic variation of pruning probability with WSS due to vessel radius (Fig 5). Vessels with small diameters with low WSS generally have low flow rates, and might be considered to be pruned, since they hardly affect the blood flow distribution in the entire vascular network. In contrast, vessels with higher shear stress carry more blood flow, and pruning of such vessels should have a greater impact on the blood flow distribution and supply more blood to the angiogenic front. It is possible that the geometry of unpruned vessels could be modified after upstream pruning [41]. To clearly answer the problem, it is needed to trace the temporal evolution of the vascular network of the same sample by using a biological model such as Zebrafish which allows to conduct live-imaging [3,42]. ECs at the angiogenic front, such as tip cells, are stimulated with VEGF for cell migration and seems to receive limited effect of blood flow. Further analysis combining such chemical effects will be warranted in future work.

## Materials and methods

### Ethics statement

All the experimental procedures involving animals were conducted in accordance with the local animal ethics committees and authorities (Regierungspräsidium Darmstadt, B2/1073) and institutional rules and regulations.

### Retina staining

For the experiment, 6 days postnatal (P6) mice were utilized. At this desired stage of development, mouse eyes were collected and fixed for 5 hours in ice-cold 2% paraformaldehyde (PFA) in PBS for 5 hours at 4°C. After that, the retina samples were dissected in PBS. Blocking/permeabilisation was applied using Blocking Buffer (BB), consisting of 1% FBS (Gibco), 3% BSA (Sigma), 0.5% triton X100 (Sigma), 0.01% Na deoxycholate (Sigma), 0.02% Na zide (Sigma) in PBS of pH = 7.4 for 2–4 hours at 4°C on a rocking platform. Then they were incubated with primary antibodies (anti-ICAM-II (BD Pharmingen, 553326, 1:100), anti-Collagen-Type IV (Collagen-IV) (Bio-RAD, 2150-1470, 1:400) and anti-VEGF164 (R&D Systems, AF-493-NA, 1:100) in 1:1 BB/PBS), overnight at 4°C on rocking platform. Retinas were then washed four times for 30 min in PBS/ 0.2% TritonX-100 (PBT) at RT and incubated with Alexa Fluor conjugated secondary antibodies (Invitrogen, 1:500) in 1:1 BB/PBS for 2hr at RT. After another four times of washing with PBT, retinas were radially cut into four lobes and flat-mounted onto slides using Fluoromount-G mounting medium (Southern Biotech, 0100-01).

### Image processing and numerical simulation

To perform the three-dimensional simulations, the confocal images were converted to three-dimensional vascular network. The images were firstly transferred to binary images in which black and white regions represent tissue and blood vessel, respectively. The vessel diameters inferred from a Collagen Type IV image commonly appear to be larger than those from a ICAM-II image, since the latter labels the basement membrane rather than the apical membrane of endothelial cells (Compare S1A and S1B Fig). We also provided zoomed figure of some local regions (green and red box) for S1A and S1B to clearly show this trend. In the present study, we extracted the centerline of each vessel and also its local vessel diameter from both the ICAM-II and Collagen Type IV images. This technique has already been used in our previous study for 1D analysis of blood flow in the zebrafish brain vasculature [43]. For all the unpruned vessels, we estimate the local vessel diameters *R*_*ICAM*_ and *R*_*Collagen*_ from the ICAM-II and Collagen Type IV images, respectively. Then, we obtained their relationship as $R_{ICAM}=\text{0}.\text{8}\text{1}R_{Collagen}-\text{0}.\text{1}\text{7}$ (see S1C Fig). This relationship is used to estimate the vessel diameters of the pruned vessels from the Collagen Type IV image. For the 3D reconstruction of the post-pruning structure, we use the ICAM-II image. Meanwhile, for the 3D reconstruction of the corresponding pre-pruning structure, the same ICAM-II image was used for reconstructing unpruned vessels. As for the pruned vessels, however, no ICAM-II signal is available, so that their vessel diameters were estimated from the Collagen Type IV image based on the above regression. Finally, the pre-pruning structure was reconstructed by adding the pruned vessels with the modified diameters from the Collagen Type IV image to the post-pruning structure. Therefore, the 3D structures of the unpruned vessels were exactly the same in the pre- and post-pruning structures, while only pruned vessels with the modified diameters were added in the pre-pruning structure (see the pink vessels in S1D Fig). In the next step, the shortest distance from the centerline to the vessel wall was calculated and then a 3D sphere with the diameter of the shortest distance was constructed on the point of the centerline. The envelope of 3D spheres at all the centerline points was used to reconstruct 3D vascular structure, and it was used as the computational domain for the present simulation. The procedure is available in the literature [17].

A signed distance function, commonly known as level-set function, was applied to represent a complex three-dimensional vascular structure and was integrated into an in-house CFD solver, which had been successfully established and applied in previous studies [17,44]. The present numerical scheme embedded complex vascular structures in a cartesian coordinate system. As a result, each grid (or pixel) contains the information whether the point is inside the blood region or the vessel wall region. The flow was assumed to be incompressible, Newtonian and steady. The governing equations for the mass and momentum conservations in the non-dimensionalized form can be expressed in Eq. (2) and Eq. (3), respectively. For the pixels corresponding to the vessel wall, we applied an artificial body force (see, the last term in Eq. (3)) so as to make the blood velocity is zero. This way, the non-slip condition on the vessel wall was achieved.


$$\begin{array}{c} \frac{\partial u_{i}}{\partial x_{i}}=\text{0}, \end{array}$$ (2)

$$\begin{array}{c} \frac{\partial u_{i}}{\partial t}+\frac{\partial \left(u_{j}u_{i}\right)}{\partial x_{j}}=-\frac{\partial p}{\partial x_{i}}+\frac{\text{1}}{Re}\frac{\partial^{\text{2}}u_{i}}{\partial x_{j}\partial x_{j}}-\eta_{u}\phi \left(u_{i}-u_{i}^{s}\right). \end{array}$$ (3)

Here *u*_*i*_ (= *u*, *v*, *w* for *i* = 1, 2, 3, respectively) were the velocity components along the *x*_*i*_ (= *x*, *y*, *z* for *i* = 1, 2, 3, respectively) directions, whereas *p* was the static pressure. The inlet bulk mean velocity (*U*_*in*_) and the diameter of inlet artery (*D*_*in*_) were considered as the characteristic velocity- and characteristic length-scales, respectively, so that the velocity components and the space coordinates were non-dimensionalized using *U*_*in*_ and *D*_*in*_. The static pressure was normalized by $\rho U_{in}^{\text{2}}$, where *ρ* is the density of the blood. The Reynolds number (Re) was defined as $\text{Re}={U_{in}D}_{in}/\nu$, where ν stands for the kinematic viscosity of the blood. The Reynolds number (*Re*) was fixed and is equal to 0.1, based on inlet bulk mean velocity (*U*_*in*_), diameter of inlet artery (*D*_*in*_) and kinematic viscosity (*ν*) reported previously [45–48]. The blood flow inside the complex three-dimensional vascular network was solved using a volume penalization method, where the effects of the complex geometry are expressed by an artificial body force acting around the interface between the fluid and solid regions. Uniform grids were generated in the whole computational domain with grid spacing $\left(\Delta_{x},\;\Delta_{y},\;\Delta_{z}\right)\approx (\text{1}.\text{5}\;\mu m,\;\text{1}.\text{5}\;\mu m,\text{1}.\text{5}\;\mu m)$.

However, to investigate the effects on the non-Newtonian viscosity, we performed an additional computation with the Carreau–Yasuda model to take the shear-thinning behavior of blood into consideration [49];


$$\begin{array}{c} \eta \left(\dot{\gamma }\right)=\eta_{\infty }+\left(\eta_{\text{0}}-\eta_{\infty }\right)[{\text{1}+{\left(\lambda \dot{\gamma }\right)}^{a}]}^{\frac{\left(n-\text{1}\right)}{a}} \end{array}$$ (4)

where *a*, *n* and *λ* are empirically determined to fit a curve between regions of constant $\eta_{\infty }$ and *η*_0_. $\dot{\gamma }$ is the shear rate. For the mouse blood, the values of these constants; $\eta_{\infty }=\text{1}\text{4}.\text{4}\text{9}\times {\text{1}\text{0}}^{-\text{3}}\text{ Pa}.\text{s}$, $\eta_{\text{0}}=\text{3}.\text{2}\text{6}\text{5}\times {\text{1}\text{0}}^{-\text{3}}\text{ Pa}.\text{s}$, λ=0.1839 s, *a* = 2.707, *n* = 0.4136 are provided in available literature [11]. S8A Fig presents the ratio of local viscosity (*η*) and the reference viscosity (*η*_0_). The maximum value of (*η*/*η*_0_) reached to 1.5 but the non-Newtonian effect appeared only near the wall of near-inlet region of the artery. For the other regions, however, the non-Newtonian effects were relatively small. S8B and S8C Fig showed the pressure distributions obtained by Newtonian and non-Newtonian blood flow simulations, respectively. In accordance with the non-Newtonian effects shown in S8A Fig, the difference between the two cases observed mainly in the artery near the inlet. Since the pruning occurred further downstream, non-Newtonian effects are considered to have minor impacts on the present results and conclusions. To confirm this, we tested the current pruning Model-2 with the local information of WSS and pressure obtained with the non-Newtonian viscosity model for the test sample of S7. The predicted pruning sites are presented in S8D Fig. We confirmed that employing the non-Newtonian viscosity model hardly affected the local haemodynamics, and therefore the pruning prediction remained unchanged from that predicted by the Newtonian model shown in Fig 3E.

### Machine learning

In developing the current machine learning models (Model-1 and Model-2), six samples (S1-S6) were used for training, and the remaining sample (S7) was used for testing out of the total seven samples. In Model-1, the location information (cylindrical coordinates, r,θ,|z|), the WSS index (α) and vessel radius (R) were used as the input data. Meanwhile, in Model-2, the location information was replaced by the local pressure information, so that all the inputs were local haemodynamic and geometric parameters. In both the models, the same network was used to predict the pruning probability at each pixel comprising the vessel wall. Although we used only six samples for training, each sample had around 1 million pixels making the vessel wall, so that the number of training points is around 6.7 million. It turned out that the number of unpruned points was about 20 times larger than the pruned points. To prevent the machine learning models from outputting a trivial solution of a zero probability for all the points, a portion of the unpruned points was randomly selected to make sure that the number of pruned and unpruned points in the training data are the same. The datapoints in unpruned vessels were reselected every 10 epochs to use all the pixels for training. The batch size was set to be 60000, which resulted in the best prediction among the other values we had tested.

As for the network structure, we first employed a feedforward neural network with 4 hidden layers and 20 nodes per layer. Leaky ReLU was used as an activation function for all the hidden layers and the sigmoid activation function for the last layer. By using the sigmoid activation function in the last layer, we were effectively constrained the network output between 0 and 1 to represent the pruning index. The loss function was set to be the binary cross-entropy between the ground truth and the model prediction. We had found that the resultant prediction accuracy was marginal. Then, we also tried a further simple network without hidden layers. In this case, the output pruning index (*pI*) was expressed as follows:


$$\begin{array}{c} pI=\text{Sigmoid}(m_{\text{1}}\alpha^{*}+{m_{\text{2}}\text{p}}^{*}+m_{\text{3}}{\text{R}}^{*}+n) \end{array}$$ (5)

Here, *m*_1_, *m*_2_, *m*_3_ and *n* were the network parameters to be optimized by training. The predicted value of *m*_1_, *m*_2_, *m*_3_ and *n* were 0.52, 0.82, -8.3 and 3.1, respectively. we could have the similar discussion on the effect of each variable on the pruning possibility using Eq. (5) as we did with Eq. (1). However, the sigmoid function applied just before the output of the network makes the contribution of each variable slightly unclear. Therefore, we proposed a further simplified model expressed by the sum of each contribution as Eq. (1). The above neural network provided a better prediction that those from more complicated networks. This might be attributed to the lack of the training data to train larger networks, and there is a possibility that the prediction accuracy could be further improved once more training data become available.

For the validation of the trained machine learning models, the network output which represents the pruning index and ranges from zero to one, has to be assessed by experimental data. We introduced the threshold value (th) for the pruning index and assumed that pruning occurred when the predicted pruning index is larger than the value. The threshold values were determined as th = 0.79 and 0.69 for Model-1 and Model-2, respectively, to maximize Intersection of Unit (IoU) of the pixels corresponding to the pruned vessels in the experiment and the model prediction (Fig 3B and 3D). Once pruned pixels with pruning index higher than the threshold value were identified, they were used to predict pruned vessels. We defined the success rate as the percentage ratio of predicted pruning sites and total pruning sites. Specifically, we defined a vessel as pruned if both ends of the vessel were connected to adjacent vessels (none of the end points are open) in the pre-pruning structure, and the perimeter of the vessel was completely covered by the pruned pixels. The vessels predicted to be pruned by the present machine learning models were shown in Fig 3E.

In present study, we mainly focused on the artery-vein (A-V) structures. However, in the mouse retina, arteries typically feed into vein segments, which could have significant changes in the flow distributions from the feeding artery. Therefore, we included a new artery-vein (A-V) structures and their extended vein-artery-vein (V-A-V) counterparts in our machine learning (ML) testing. Since the image of Collagen Type IV for this structure is not available, our aim was to assess the impacts of the two different configurations (A-V and V-A-V structures) on the local hemodynamics and the resulting prediction of pruning sites for the same sample. The predicted pruning sites by ML model-2 for both A-V and V-A-V configurations are plotted in S9A and S9B Fig. The orange circles, i.e., totally 20 vessels, indicate predicted pruning sites in both the A-V and V-A-V configurations, while only one vessel with a black circle was not identified as a pruning vessel in the V-A-V configuration. Considering that the two structures, albeit extracted from the same sample, are quite different, it is amazing that the prediction of pruning vessels is quite robust in the way to extract the vessel structure. This is mainly due to the fact that the present model is based on the normalized blood pressure and the relative difference of the wall shear stress to its local average. This consistency supports the robustness of the present approach.

## Supporting information

S1 Fig 3D reconstruction of pre- and post-pruning vessel structures. (A) The vessel centerlines extracted from (A) a Collagen Type IV image and (B) a ICAM-II image. (C) The relationship between the local radius estimated from the Collagen Type IV and ICAM-II images, (D) 3D structure from post-pruning image (white vessels) with pruned vessels (red vessels).
(TIF)

S2 Fig 3D structures of the vascular network with pruned vessels used for training (sample no. S1-S7). Pruning locations were shown in pink color.
(TIF)

S3 Fig The distribution of experimental pruning probability (*pp*__*exp*_) over the range of WSS index (*α*) for different window sizes. (TIF)

S4 Fig Effect of training samples data on prediction accuracy using Model-2. **(A)** Actual pruning locations highlighted by orange circles for sample S1 observed in the present experiment. **(B)** Predicted pruning locations using 6 training samples (a2 to a7). **(C)** Predicted pruning locations using 5 training samples (a2 to a6). **(D)** Predicted pruning locations using 4 training samples (a2 to a5). In panels B, C, and D, orange, black and red circles indicate successfully predicted, unpredicted and extra-predicted sites, respectively.
(TIF)

S5 Fig Validation of machine learning models. **(A)** Comparison of vessel pruning predicated by machine learning models (Model-1 and Model-2) with experimentally identified pruning sites (experiment) from sample S1. Orange, black and red circles illustrate the predicted pruning sites, unpredicted pruning sites and extra predicted sites, respectively. **(B)** Threshold values for the pruning probability in Model-1 and Model-2.
(TIF)

S6 Fig Comparison between pruning locations for sample 7 observed in the experiment and those predicted by the present machine learning models. **(A)** Actual pruning locations highlited by orange circles for sample S7 observed in the present experiment. **(B)** Pruning locations predicated by Model-2 based on the local pressure, radius and WSS index (*α*). **(C)** Pruning locations predicated based on only the local radius. **(D)** WSS index (*α*) distribution at the four different regions, where new unpredicted and extra predicted sites emerged. In S6B and S6C Fig, orange, black and red circles indicate successfully predicted, unpredicted and extra-predicted sites, respectively. The circles with broken lines in S6C Fig show newly emerged unpredicted and extra-predicted sites when the local haemodynamics information is not used for the prediction.
(TIF)

S7 Fig Comparison between pruning locations for sample S2 (first row) and S3 (second row) observed in the experiment and those predicted by the present machine learning model. **(A)** Actual pruning locations highlighted by orange circles for sample S2 observed in the present experiment. **(B)** Pruning locations predicated for sample S2 by Model-2 based on the local pressure, radius and WSS index (*α*). **(C)** Pruning locations predicated for sample S2 based on only the local radius. **(D)** Actual pruning locations highlighted by orange circles for sample S3 observed in the present experiment. **(E)** Pruning locations predicated for sample S3 by Model-2 based on the local pressure, radius and WSS index (*α*). **(F)** Pruning locations predicated for sample S3 based on only the local radius.
(TIF)

S8 Fig Effects of non-Newtonian viscosity on the local haemodaynamics and the pruning prediction. **(A)** the ratio of the local viscosity (*η*) calculated by the non-Newtonian viscosity model (Carreau–Yasuda model) and the reference constant viscosity (*η*_0_) used in the present Newtonian calculation. The maximum value of (*η*/*η*_0_) reached to 1.5, while the non-Newtonian effect appeared only near the inlet of the artery. S8B and S8C Fig showed the pressure distributions for Newtonian and non-Newtonian flows, respectively. S8D Fig presented the predicted pruning sites by Model-2 based on the local haemodynamic parameters obtained by the non-Newtonian viscosity model for the test sample of S7. The prediction remained unchanged from that by the Newtonian viscosity model shown in Fig 3E.
(TIF)

S9 Fig Comparison between Pruning locations for artery-vein (A-V) structure and their extended counterparts vein-artery-vein (V-A-V). (A) Predicted pruning locations in artery-vein structure using ML model (B) Predicted pruning locations in vein-artery-vein (V-A-V) structure only on same segment as A-V structure using ML model. Orange circles indicate the matched pruning sites in A-V and V-A-V structure. Black circle shows the unmatched pruning site.
(TIF)

S1 Table Different fitting and corresponding R-square value. (DOCX)
