# Supplementary material for: Complex relationship among vessel diameter, shear stress and blood pressure controlling vessel pruning during angiogenesis
Source: PLoS Comput Biol. 2026 Jan 12;22(1):e1013565. doi: 10.1371/journal.pcbi.1013565 (PMC13561423; doi:10.1371/journal.pcbi.1013565)
Supplement: Supplementary file 1 [file pcbi.1013565.s010.docx]

**S1 Table** Different fitting and corresponding R-square value

| **Fitting** | **R-square** |
| --- | --- |
| Linear | 0.934 |
| Polynomial (O^2^) | 0.955 |
| Polynomial (O^3^) | 0.989 |
| Polynomial (O^4^) | 0.990 |
| Polynomial (O^5^) | 0.990 |
| Polynomial (O^6^) | 0.990 |
